# The promotion mechanism of prebiotics for probiotics: A review

**DOI:** 10.3389/fnut.2022.1000517

**Published:** 2022-10-05

**Authors:** Siyong You, Yuchen Ma, Bowen Yan, Wenhui Pei, Qiming Wu, Chao Ding, Caoxing Huang

**Affiliations:** ^1^Co-Innovation Center for Efficient Processing and Utilization of Forest Resources, College of Chemical Engineering, Nanjing Forestry University, Nanjing, China; ^2^Department of Food Science and Technology, National University of Singapore, Singapore, Singapore; ^3^Food Science and Technology Center, National University of Singapore (Suzhou) Research Institute, Suzhou, China; ^4^Nutrilite Health Institute, Shanghai, China; ^5^Department of General Surgery, Nanjing Drum Tower Hospital, The Affiliated Hospital of Nanjing University Medical School, Nanjing, China

**Keywords:** prebiotics, probiotics, mechanism, intestinal flora, promotion

## Abstract

Prebiotics and probiotics play a positive role in promoting human nutrition and health. Prebiotics are compounds that cannot be digested by the host, but can be used and fermented by probiotics, so as to promote the reproduction and metabolism of intestinal probiotics for the health of body. It has been confirmed that probiotics have clinical or health care functions in preventing or controlling intestinal, respiratory, and urogenital infections, allergic reaction, inflammatory bowel disease, irritable bowel syndrome and other aspects. However, there are few systematic summaries of these types, mechanisms of action and the promotion relationship between prebiotics and probiotic. Therefore, we summarized the various types of prebiotics and probiotics, their individual action mechanisms, and the mechanism of prebiotics promoting probiotics in the intestinal tract. It is hoped this review can provide new ideas for the application of prebiotics and probiotics in the future.

## Introduction

Since entering the 21st century, the health of intestinal flora has become a topic of concern among many scientists. Compared with the symbiotic flora in other parts of the human body, the number of intestinal flora is larger and more complex. The total number of adult intestinal flora is approximately 3.9 × 10^13^ in colon, slightly more than the total number of human cells (1). Intestinal flora helps the host digest nutrients in food and participates in systemically physiological activities of the human body, which are closely related to the human health. According to its effect on the human body, we divide intestinal flora into three functional categories: probiotics, neutral bacteria, and pathogenic bacteria (2). In healthy individuals, these compete and restrict with each other, maintaining a normal dynamic balance.

The intestinal tract, which has been described as the second brain of the human body, plays an important role in health. The intestinal flora interacts with the human body, helping the body to digest and absorb nutrients from food, metabolize toxic waste produced in the intestine, and produce functional substances necessary for life, such as amino acids, vitamins, short-chain fatty acids (SCFAs) and other substances (3, 4). If the intestinal flora is disturbed, it will affect human health and even cause diseases such as obesity, diabetes, irritable bowel syndrome and colon cancer, so it is important to maintain its balance (5–7). Therefore, in order to keep the intestinal flora in a healthy state, it is necessary to use external forces to regulate the number of probiotics and pathogenic bacteria.

Probiotics are living microorganisms that provide benefits to host health by colonizing the body when given in sufficient amounts. Probiotics can adjust the structure of human intestinal microorganisms and inhibit the colonization of pathogenic bacteria in the intestine. It addition, probiotics show ability to help the body build a healthy protective layer of intestinal mucosa, enhancing the intestinal barrier effect and improving immunity (8, 9). According to the characteristics of probiotics, we need to understand their mechanism of action on the human body and promote their growth and reproduction.

The growth and reproduction of probiotics cannot be achieved without the promotion of prebiotics. Prebiotics are ingredients, mostly polysaccharides, that cannot be digested and absorbed by the human body, which can contribute to the growth or reproduction of active microorganisms in the host (10). Prebiotics have the function of improving the regulation of immunity, resisting pathogens, influencing metabolism, increasing mineral absorption, and enhancing health (11). Prebiotics usually refer to certain polysaccharides, oligosaccharides, microalgae, and natural plants, with a wide range of sources. Emerging prebiotics are mainly found in algae, fruit juice, peels, seeds, traditional Chinese medicine, and microorganism involving polysaccharides, polyphenols, and polypeptide polymers.

Most previous studies have focused on the health benefits of prebiotics and probiotics (12, 13). However, systematic studies on the types of prebiotics and probiotics, their mechanisms and the relationship between them are lacking. Therefore, this paper provides a comprehensive description of the common types of prebiotics, the functional sources of emerging ones, and their mechanisms of prebiotics in the intestine. In addition, the types, functions, and applications of probiotics are described, and the mechanisms of probiotic effects on the human body are described in detail. Furthermore, this review also focuses on the promotion mechanism between prebiotics and probiotics. It is hoped that this review can help researchers understand the relationship between prebiotics and probiotics and provide ideas for human health, especially the balance of intestinal flora.

## The concept of prebiotics

The definition of a prebiotic was first introduced in 1995 as an indigestible component of the body. It is an ingredient that cannot be digested by the human body, which can resist gastric acid and is not decomposed by mammalian enzymes and absorbed by the gastrointestinal tract. Prebiotics are fermented by the intestinal flora and selectively stimulate a certain number of bacteria present in the colon, thereby altering their growth and activity to beneficially affect the host (14). In 2004, Gibson et al. (15) proposed that prebiotics are ingredients that can be selectively fermented and specifically alter the composition and activity of beneficial host health flora in the intestine, which is termed as “bifidogenic factors”. In 2016, the International Scientific Association for Probiotics and Prebiotics redefined prebiotics as substances that can be selectively used and transformed by the host intestinal flora under the premise that they are beneficial to host health. The new definition of prebiotics includes non-carbohydrates, and the site of action is not limited to the gastrointestinal tract, nor is its type limited to food (16).

## Types of prebiotics

Previous studies have considered prebiotics to be oligosaccharide carbohydrates, of which mainly include xylooligosaccharides (XOS), galacto-oligosaccharides (GOS), lactulose and inulin and its derived fructose-oligosaccharides (FOS) (17–20). However, recent studies have found that prebiotics include not only carbohydrates, but also other non-carbohydrates that meet the prebiotic criteria, such as polyphenols isolated from fruits such as black raspberries (21) and blueberries (22). With the continuous optimization of the prebiotic preparation process, new prebiotic species continuously are being developed, mainly including polysaccharides, polyphenols and polypeptide polymers, which have broad research prospects.

### Galacto-oligosaccharides

**Figure 1:**
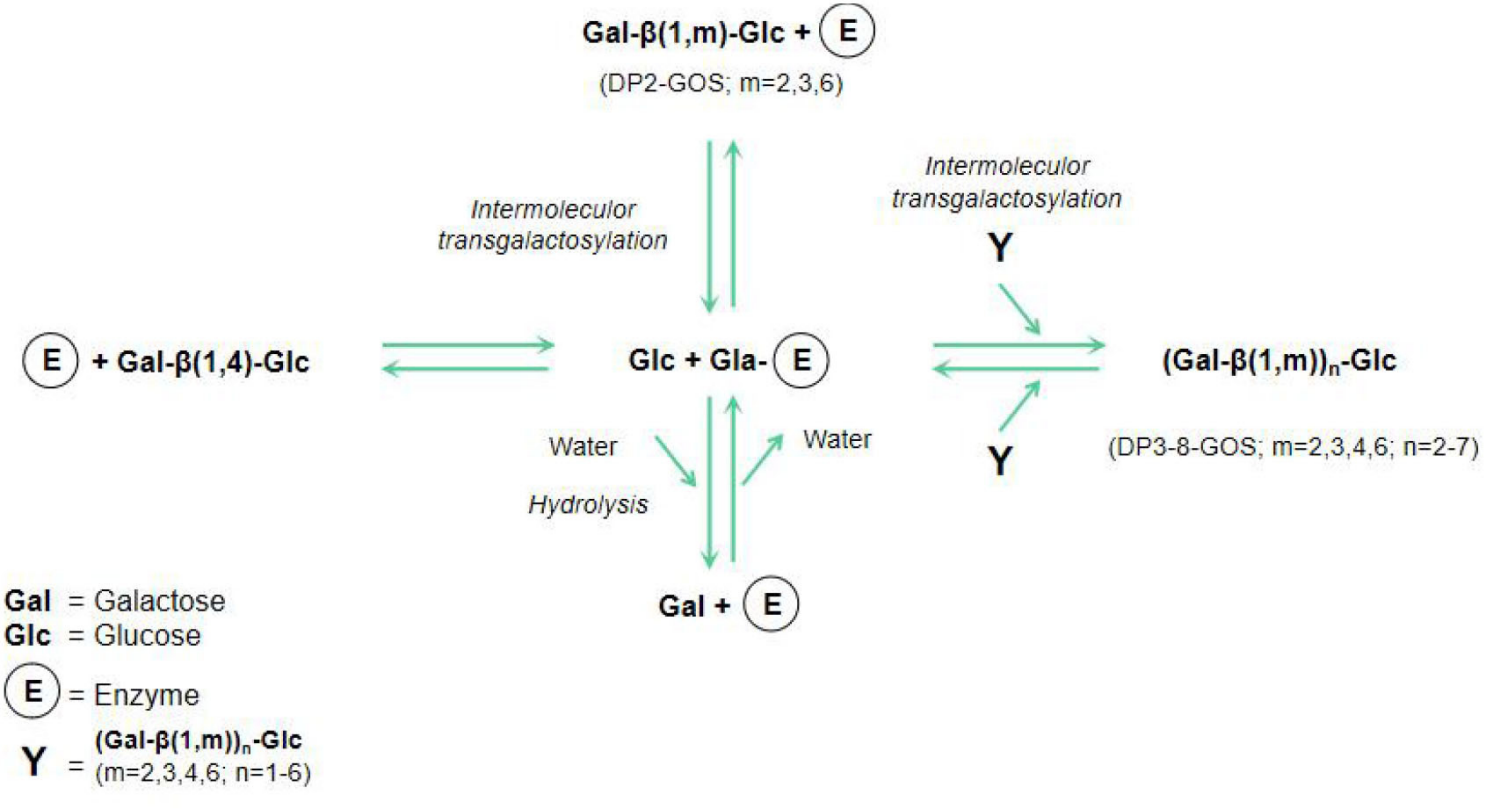
Schematic model of lactose hydrolysis and GOS synthesis (23).

GOS, a new functional substance with natural properties, are not easily digested and absorbed by the body. GOS are composed of two to eight sugar units, one of which is a terminal glucose, and the rest are galactose and disaccharides (containing two galactose units) (23). An important feature of GOS is their hybrid structure, as evidenced by the different glycosidic linkages between its glucose and galactose or between the degree of polymerization (DP) and galactose molecules (24). Figure 1 shows the schematic model of lactose hydrolysis and GOS synthesis from glucose and galactose by enzyme.

Several studies have evaluated the toxicity and genotoxicity of GOS, which have proven that GOS are a very safe food ingredient (24–28), and various regions, including the United States, Japan, and the European Union, have granted official safety approval for them (29). As one of the most common and widely used prebiotics, GOS have many healthy properties, such as regulating the balance of human colon microbiota and promoting the proliferation of *Bifidobacterium* in the intestine (29, 30). Currently, GOS are mainly used in formula and infant milk powder. When infants cannot get human milk, formula enriched with GOS or a mixture with FOS can effectively replace human milk and alter infants' intestinal flora (10, 31). An experiment with 35 healthy full-term infants revealed that *Bifidobacterium* abundance increased significantly after being fed infant formula supplemented with GOS, while a decrease in microbiota alpha-diversity was apparent GOS (OM55N). In addition, their fecal pH and SCFAs patterns were similar to those of control infants, suggesting that GOS stimulate indigenous *Bifidobacterium* growth and establish microbiota similar to that of breast-fed babies (32). That experiment's findings were consistent with those of Fanaro et al. (33), who presented multiple studies of over 400 preterm and term infants, where probiotic mixtures (short-chain GOS and long-chain FOS) effectively stimulated the growth of *Bifidobacterium* and lactic acid bacteria (LAB), reduced pathogen growth, and made the stool characteristics of the experimental subjects consuming GOS-containing infant formula consistent with those of breast-fed infants.

### Inulin-type fructans

In addition to GOS, inulin-type fructans are common carbohydrates, which can meet the criteria for prebiotics. Inulin-type fructans are polymers made from fructose linked to terminal α-linked glucose by β-2,1 bond. The longer chain is inulin, which has a DP of 2–60, and the shorter chain is oligofructose/ FOS, which has a DP of 2–8 (34). Several studies have shown that inulin-type fructans can promote the growth of *Bifidobacteria, Anthobacteria* and LAB (35).

Inulin, a water-soluble storage polysaccharide, is a non-digestible carbohydrate (fructan-type) (36, 37). Inulin has been part of the daily diet of humans for centuries and is widely found in ~36,000 plant species, of which chicory root is considered to be the richest source of inulin (38, 39). Inulin consists of a linear chain of β-2,1-linked d-fructofuranose molecules terminated at the reducing end by a sucrose-type linkage by a glucose residue. The presence of β-()-D-frutosyl fructose bonds between the fructose unit and the isomeric carbon of inulin makes it difficult for inulin to be absorbed and digested by the human small intestine, but it can be fermented by the intestinal flora of the human large intestine (40, 41).

Inulin is widely used in food processing as a multi-purpose ingredient with the following main functions: (i) replace fats or carbohydrates to give good flavor to food, but it only provides approximately one-third of the energy and is much less sweet than sucrose (42, 43); (ii) promote the absorption of minerals (e.g., calcium, magnesium, and iron) (44, 45); (iii) relieve constipation, prevents gastrointestinal diseases, and stimulate the immune system (46); and (iv) as a prebiotic with bifidogenic effect, effectively stimulate the development and metabolism of *Bifidobacterium* and *Lactobacillus* in the colon and increase the activity of intestinal microorganisms (38, 45). In addition, the relative abundance of *Anaerostipes, Faecalibacterium* and *Lactobacillus* increased and that of Bacteroides decreased after inulin supplementation (46).

Another well-known inulin-type fructan, FOS, are found in a variety of natural plants, such as bananas, wheat, garlic and onions. They are low-calorie, non-digestible carbohydrates whose DP is <10 and are also a common prebiotic in the food industry (47–50). FOS have a variety of beneficial physiological effects, such as being low in carcinogenicity, improving mineral absorption in the intestine, and lowering serum cholesterol levels, triacylglycerols, and phospholipids (51). Among them, the prebiotic activity of FOS in gastrointestinal digestion is the focus of our focus. FOS improve the body's intestinal flora, relieve constipation, reduce the risk of heart disease and certain cancers, improves lipids in hyperlipidemia, inhibit the production of intestinal putrefactive substances, and allow the body's digestive system to function more healthily (51–54).

### Emerging prebiotics

With the advancement of technology, the preparation methods of prebiotics have been optimized. In addition, various new prebiotic species (mainly including polysaccharides, polyphenols, and polypeptide polymers) have been developed (55). Emerging prebiotics are found mainly in algae, fruit juices, fruits and their waste, herbal medicines, and microorganisms from a wide range of sources. Although the knowledge of these prebiotics is not as good as that of FOS and GOS, their potential deserves to be studied in depth and has a promising future. In order to overview the prepared emerging prebiotic in recent years, the functions of polysaccharides, polyphenols and peptide polymers were listed in Table 1.

**Table 1:** Different kinds of emerging prebiotics.

**Prebiotic**	**Component**	**Source**	**Function**	**References**
Polyphenol	Blueberry polyphenol extract	Blueberry	Reduce weight and normalize lipid metabolism	(22)
	Wine grape seed flour	Grape seed	Intestinal permeability is enhanced, and adipocyte gene expression is altered to inhibit high-fat-induced obesity and inflammation.	(56)
	Orange albedo	Orange	Stimulates the growth, reproduction, and metabolism of *Lactobacillus acidophilus* and *Lactobacillus animalis*	(57)
	Catechin and punicalagin	Fermented pomegranate juice	Increases antioxidant capacity and improves survival of lactic acid bacteria	(58)
Polypeptide polymers	Poly-gamma-glutamate (PGA)	Bacillus fermentation	Increases abundance of *Lactobacillus* and reduces abundance of *Clostridium*, helping to regulate the intestinal microbiota.	(59)
Polysaccharides	Algae polysaccharides	Algae	Improves the activity of some beneficial flora and stimulates the production of functional metabolites in the intestinal microbiota.	(60)
	Lotus seed resistant starch (LRS3-20%)	Lotus seed	Shows high probiotic activity against *Bifidobacterium* and *Lactobacillus acidophilus*.	(61)
	Longan pulp polysaccharides	Logan	Promotes the growth of *Lactobacillus plantarum, Lactobacillus bulgaricus* and *Lactobacillus fermentum*	(62)

## Mechanism of action of prebiotics

**Figure 2:**
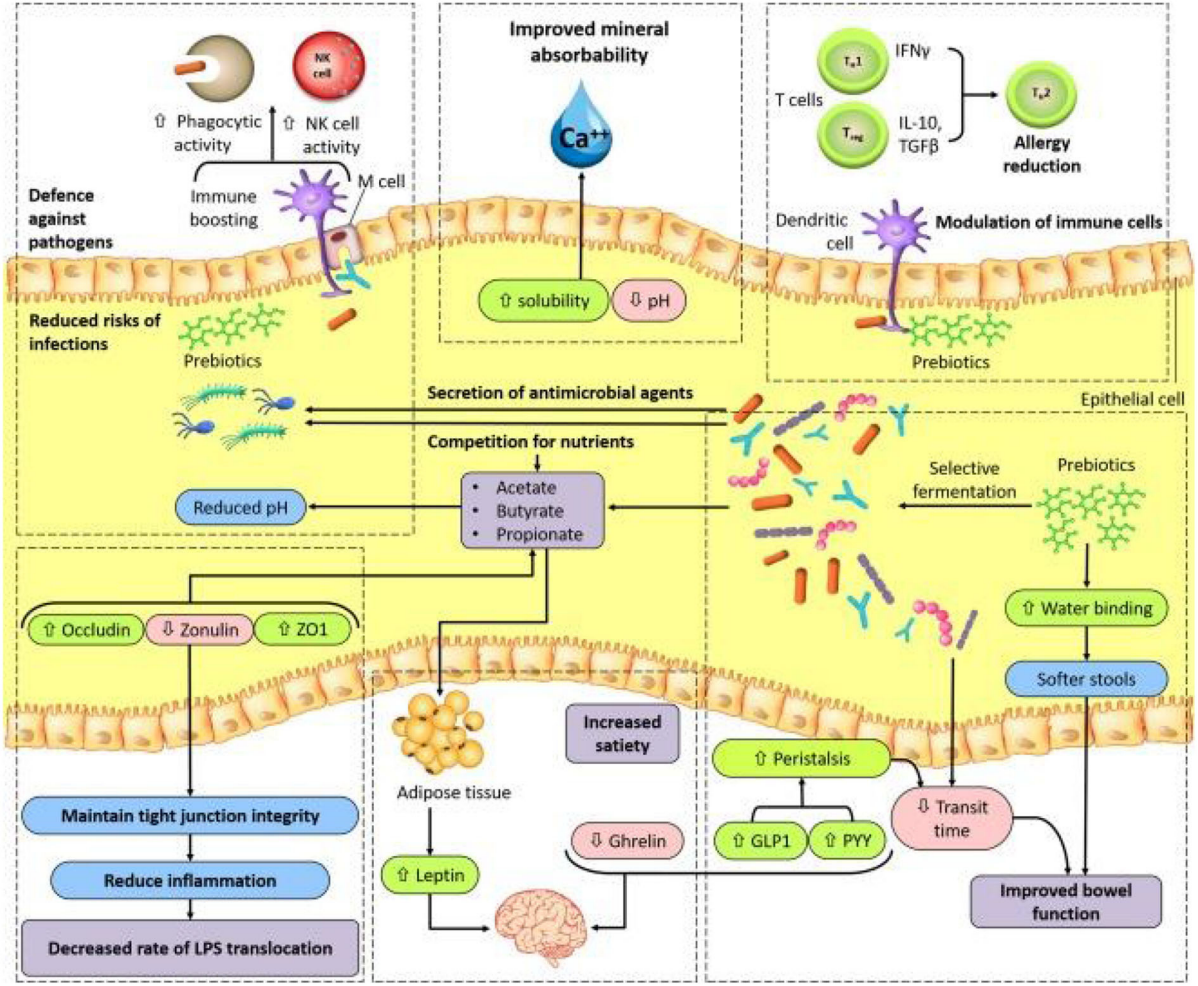
A model for possible mechanisms of prebiotic benefits to human health (66). GLP1, glucagon like peptide1; M cell, microfold cell; NK, natural killer; PYY, peptide YY; TGFβ, transforming growth factor-β; TH1, TH2, type 1 T helper, type 2 T helper; Treg, regulatory T; ZO1, zonula occludens 1.

**Figure 3:**
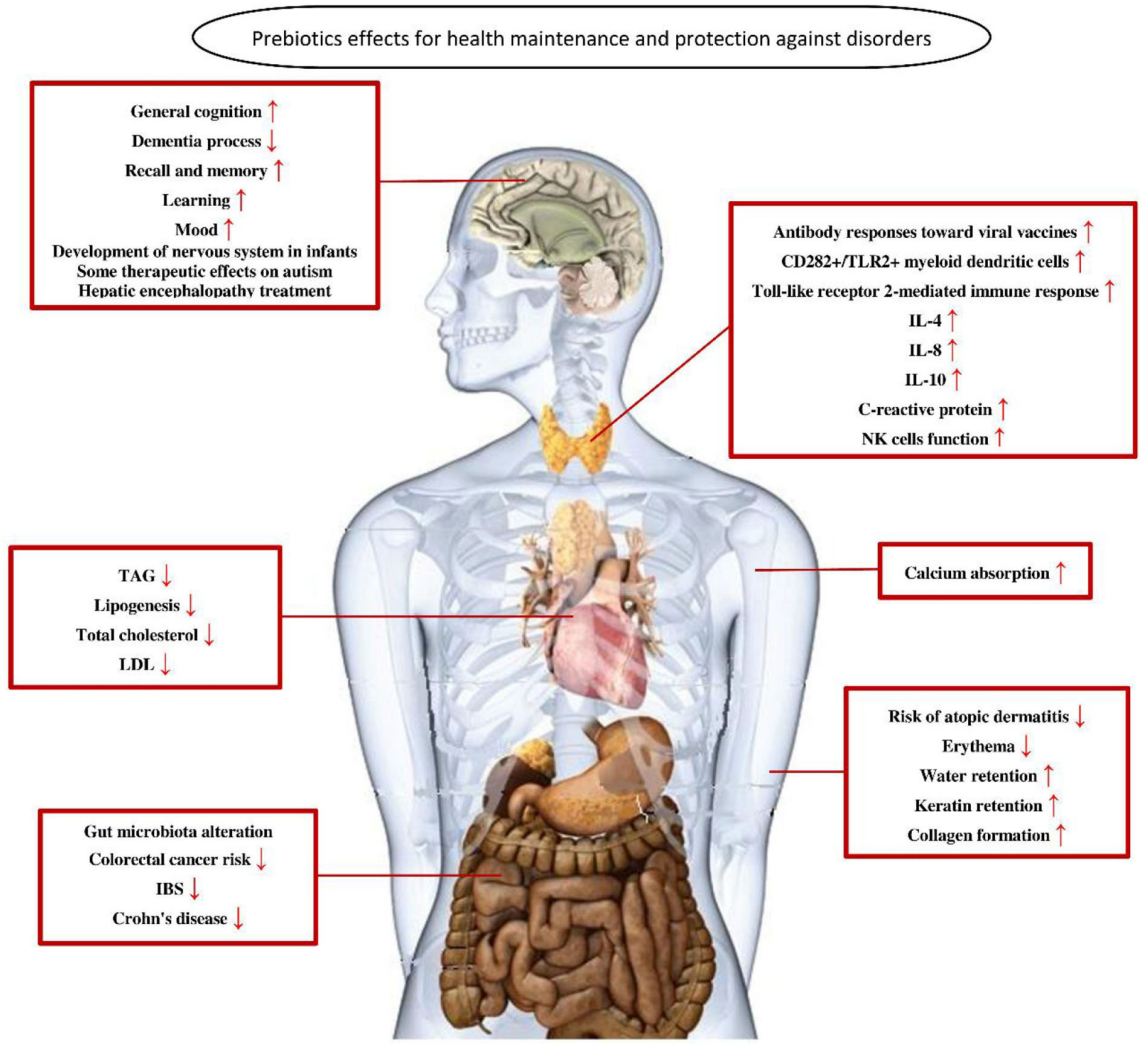
The role of prebiotics in maintaining health and preventing disorders (68). TAG, triacylglycerol; LDL, low-density lipoprotein; IBS, irritable bowel syndrome; IL-4, interleukin 4; IL-8, interleukin 8; IL-10, interleukin 10.

In general, in the human intestine, the lack of enzymes that hydrolyze the polymer bonds of prebiotics allows them to remain in the gastrointestinal tract to resist digestion in the small intestine. The human body then transports these prebiotics intact to the large intestine, where they are degraded by the intestinal flora and selectively fermented to produce certain secondary metabolites, which are absorbed by the intestinal epithelium or transported to the liver through the portal vein and can have beneficial effects on host physiological processes, exerting effects such as regulating immunity, resisting pathogens, improving intestinal barrier function, increasing mineral absorption, and lowering blood lipid levels (29, 63, 64). The most abundant SCFAs in the intestine are metabolized by beneficial bacteria, including acetate, butyrate and propionate, which are beneficial to maintaining intestinal and systemic health (65). Moreover, a specific advantage of prebiotics is referred to their promotion for the growth of target microorganisms. After consumption of specific prebiotics (e.g., inulin, FOS, and GOS), they can promote the growth of beneficial flora to compete with other species by protecting or promoting the production of beneficial fermentation products (66, 67). The possible mechanisms by which prebiotics promote health benefits in humans are shown in Figure 2. In addition, prebiotics have a protective effect not only on the gastrointestinal system but also on other parts of the body, such as the central nervous, immune, and cardiovascular systems (Figure 3).

## Overview of different probiotics

**Figure 4:**
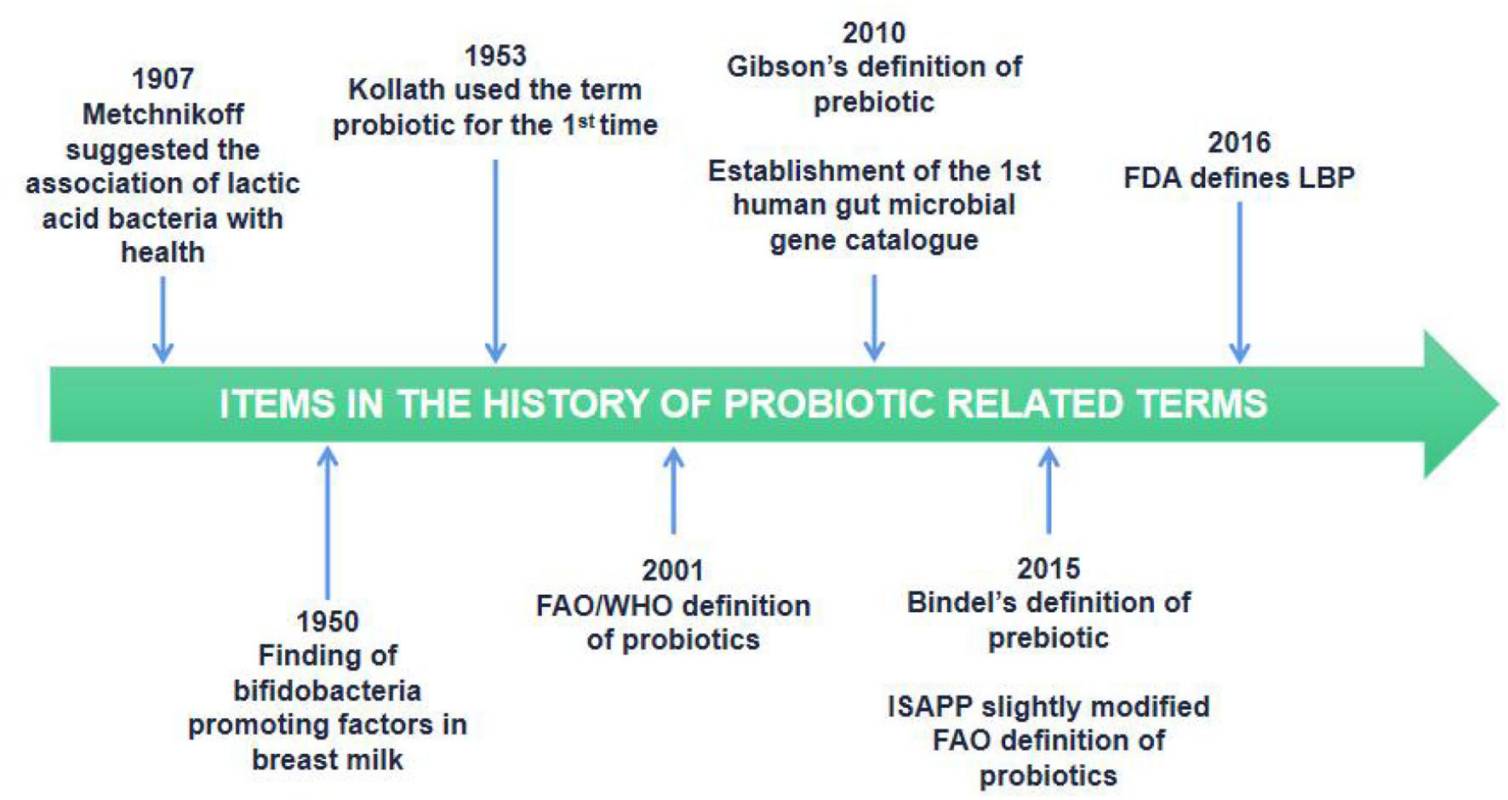
Timeline of selected items in the history of probiotic related terms.

The concept of traditional probiotics was originally born based on the observations of Elie Metchnikoff in 1907, who found that the health and increased longevity of older Bulgarians was linked to their daily intake of fermented dairy products rich in LAB, such as yogurt. Metchnikoff noted that bacteria may have a beneficial effect on the natural gut microbiota. Since then, probiotics have been symbolized as bacteria that benefit the health of the host. Over time, the definition of probiotics has been largely modified (Figure 4) (69).

According to the definitions of probiotics by Food and Agriculture Organization of the United Nations and World Health Organization, probiotics are live strains of microorganisms that have been rigorously screened and, when properly ingested, can exert beneficial effects on the health of those who consume them (70). Probiotics can perform vastly positive functions on the human body, such as regulating human intestinal health, maintaining the balance of microflora, regulating human immune function, helping the body to better digest and absorb food residues, improving blood lipid and blood sugar metabolism, and treating and alleviating lactose intolerance, which has a positive impact on human health (71–73). To achieve good health benefits, probiotics must be able to grow in the food product and survive in sufficient numbers until they reach their final destination, the intestine. Therefore, it is clear that the factor that must be considered in the selection of probiotics is their adhesion to the intestinal mucosa and intestinal epithelial cells (74).

**Figure 5:**
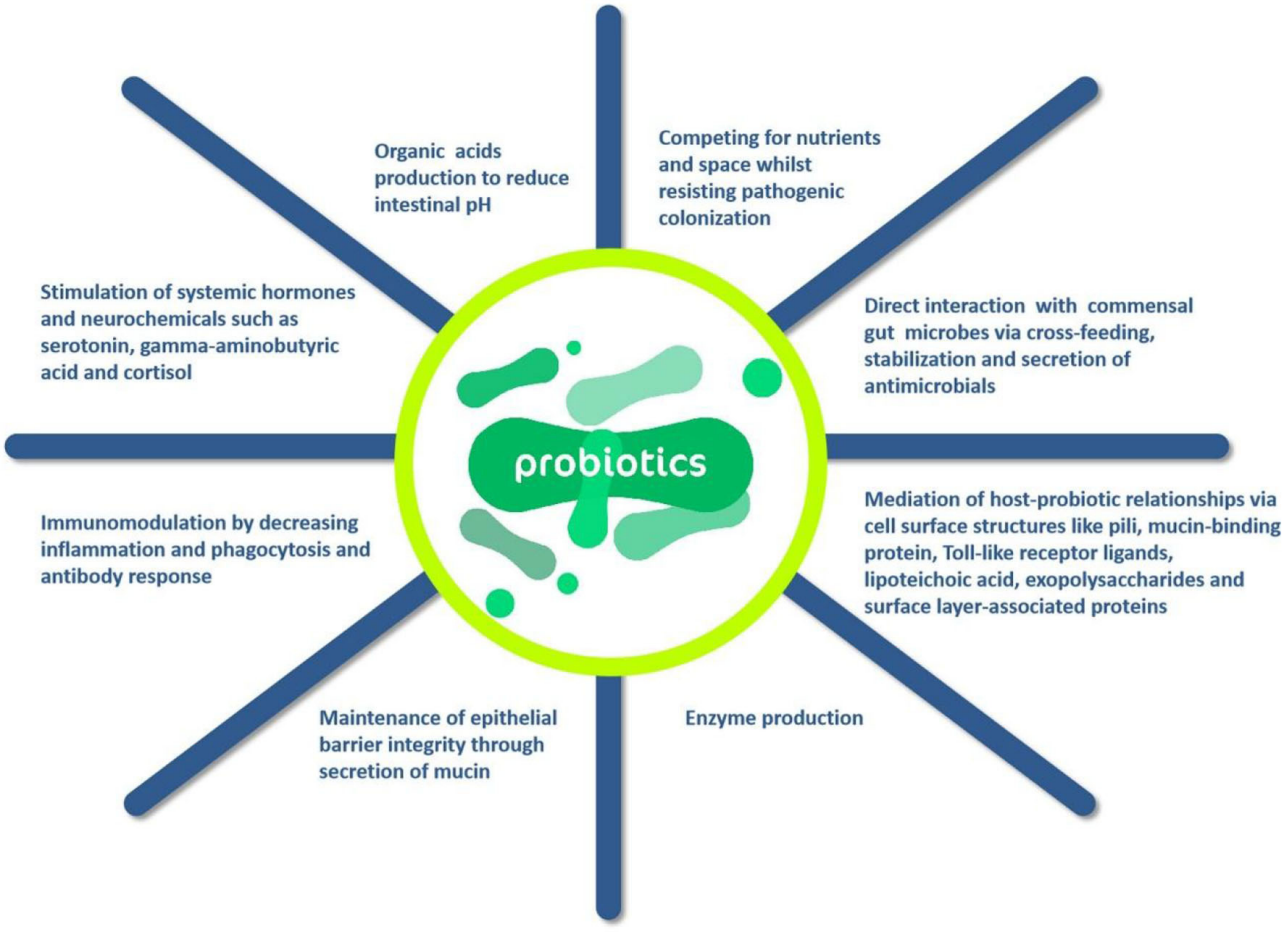
Action mechanisms of probiotics (66).

Probiotics exert beneficial effects on the body through four main mechanisms: prevention of potential pathogens and inhibition of their growth, improvement of the barrier function of the gut, immunomodulation of the body, the production of neurotransmitters can modulate the host (75). Oelschlaeger (76) found that probiotics can directly affect or act on other microbial products, host products, or food ingredients by modulating the host's immune system. However, a point of concern is that the health improvement of probiotics is dependent on the probiotic flora added, and what function the probiotic performs depends on its metabolic characteristics and surface molecules or secreted components of the cells of these microorganisms. Furthermore, whole composition, such as DNA and peptidoglycan, may play a very important role in the efficacy of probiotics. It should be mentioned there is no single strain can provide all the above-mentioned probiotic properties of probiotics. According to the action mechanisms of probiotics in Figure 5, it can be known that probiotics can provide a variety of means to enhance host immunity, which in turn directly affects immune cells and other host cells. In addition, the antimicrobial component substances and cross-feeds can be produced by probiotics, which is generally produced from the combination of multiple action of microorganisms.

## Types of probiotics

### Lactobacillus

Probiotics have a wide distribution range and a variety of species, which can be broadly classified into three major groups: *Lactobacilli, Bifidobacteria* and others. At present, most studies on probiotic species are aimed at the LAB group, which is the most representative probiotic in the LAB group. *Lactobacillus* is an important probiotic in the study of human intestinal microorganisms, which is closely related to human health; it not only synthesizes essential vitamins and amino acids and promotes mineral absorption, but importantly, it can achieve the effect of improving intestinal microecology by inhibiting the growth of harmful microorganisms (77). In addition, SCFAs, an important metabolite of *Lactobacillus*, help maintain the normal physiological function of the colon and the morphology of the colonic epithelium, as well as promote the growth and reproduction of *Lactobacillus*, thus reducing the number of *Escherichia coli* in the intestine (78).

*Lactobacillus* has a significant positive effect on host growth, especially by improving body weight and size. For example, Liu et al. (79) found that *Lactobacillus plantarum* (which is termed as *Lactiplantibacillus plantarum* according to the updated microbial taxonomy) *ZJUFT17* (T17), isolated from traditional Chinese sour dough, could act as a potential probiotic with anti-obesity or weight loss properties and could improve systemic inflammation and insulin resistance mediated by intestinal microbiota. Specifically, the weight gain, energy intake and serum lipids of high-fat diet induced mice could be inhibited when 24 × 10^8^ cfu of T17 was administered for mice for 10 weeks. Li et al. (80) investigated the use of soymilk fermented with *Lactobacillus plantarum* HFY01 for weight reduction and lipid-lowering in mice with HFD-induced obesity, and the results were similar. *Lactobacillus plantarum* HFY01 fermented soymilk reduced body fat percentage and liver index in obese mice and strongly inhibited high-fat diet-induced obesity, showing good utilization potential.

*Lactobacillus* influences microbial interventions to maintain or improve microbial balance in the host environment and to inhibit pathogen invasion. Synergistic interactions between LAB and endogenous commensal flora are an important factor in restoring microbial endostasis (81). For example, LAB in sour dough can be used in combination with plant- and/or animal-based ingredients. Due to the different mechanisms of action and ideal symbiotic activities, the functional characteristics of the whole combination can be enhanced, and it has good flavor and nutritional value (82). In addition to the synergistic effect with commensal flora, LAB produce compounds with antimicrobial effects in the defense against pathogens by enhancing the epithelial barrier function of the intestine (83, 84).

### Bifdobacterium

*Bifidobacterium* is a genus of Gram-positive specialized anaerobic bacteria that is often bifurcated at the end, which is the origin of its name (85). It is a physiological bacterium that exists in the human body and is a very important group of probiotics for humans. *Bifidobacterium* can adapt to the anaerobic intestinal life, reproduce and metabolize in the middle and end of the small intestine and large intestine, and secrete bifidogenic factors with probiotic effects to regulate intestinal health (86, 87). Currently, Bifidobacterium includes 32 species and 9 subspecies, 14 of which have been isolated from humans (88).

The physiological functions of Bifidobacterium are mainly as follows: (i) Similar to other LAB, *Bifidobacterium* can restrain the growth of pathogenic bacteria, so as to maintain the balance of normal intestinal bacterial flora and inhibit pro-inflammatory cytokines (89, 90). Related experiments have also demonstrated that *Bifidobacterium* can protect against intestinal barrier dysfunction both *in vitro* and *in vivo*. This protective effect is associated with inhibition of pro-inflammatory cytokine secretion and vimentin release and improvement of intestinal tight junction integrity (91). (ii) *Bifidobacterium bifidum* synthesizes vitamins and amino acids in the intestine and increases calcium bioavailability, and it is thought to improve bone health (92, 93). (iii) *Bifidobacterium bifidum* has anti-tumor effects. Shimizu et al. successfully produced a strain of *Bifidobacterium* longum that secretes C-CPE-PE23 and can selectively localize and proliferate in tumors. The isolated *Bifidobacteria* were specifically distributed in the tumors of mice with breast cancer and significantly inhibited tumor growth without serious side effects such as weight loss or liver and kidney damage, and the experimental results suggest that *Bifidobacteria* can be special carriers of anti-cancer proteins against malignant tumors (94).

### Other bacteria species

In addition to *Lactobacillus* and *Bifidobacterium*, Gram-positive parthenococci such as *Enterococcus* are frequently used in the food industry today. The ability of Enterococcus strains to survive, compete and attach to host cells in the intestine is a key feature as a probiotic. In addition, Enterococcus is highly resistant to a wide range of pH and temperature; this is attributed to its strong bacteriocin production capacity, which can be used as a natural antimicrobial agent in the food industry (95).

*Saccharomyces cerevisiae* is a well-known non-pathogenic and selective probiotic that is now used in the commercial production of probiotic foods. *Saccharomyces boulardii*, for example, has been extensively studied for its probiotic action and is commonly used to treat digestive disorders such as diarrhea symptoms, especially when used as an adjunct to antibiotic therapy. Furthermore, when passing through the digestive tract, *Saccharomyces boulardii* has a higher survival capacity compared to other probiotics, helping to maintain the balance of the normal microflora of the intestinal tract. It also has immunomodulatory effects, acting to fine-tune immunological pathways during pathogenic infections or chronic diseases (96–98).

In addition to the enterococci and yeasts mentioned above, other categories of probiotics that are more common are *Bacillus* species, *Streptococcus* species, and *E. coli*. Based on recently published work, the roles and applications of some common probiotics were summarized in Table 2.

**Table 2:** Functions and applications of probiotics.

**Strain**	**Function**	**Application**	**References**
*Bifidobacteria*	The exopolysaccharides produced have antioxidant, anticancer, antibacterial, and immunological activities	Used as a starter culture for fermented foods	(99)
*Lactobacillus casei*	Prevention or treatment of diseases that disrupt the intestinal microbiota	Dairy fermentation	(100)
*Bifidobacterium adolescentis*	It reduces the inflammation of spleen and brain, and changes the microbiota of cecum and colon	Medicine and clinic	(101)
*Lactobacillus acidophilus*	Reduces cytokines to relieve inflammatory bowel disease, alleviate cancer, modulate immunity, lower cholesterol and relieve diarrhea	Medicine and clinic	(102)
*Bacillus coagulans*	It can regulate the balance of intestinal microbiota, promote the metabolism and utilization of nutrients, improve immunity, and has the characteristics of high temperature resistance, acid resistance, and bile resistance	Medicine and animal husbandry	(103, 104)
*Bacillus subtilis*	Improved growth, nutrition, immunity and disease resistance of aquatic species	Aquaculture	(105)
*Lactobacillus rhamnosus*	Has the ability to fight against pathogenic bacteria and fungi in the genitourinary tract, preventing the recurrence of urinary tract infections in postmenopausal women	Fermentation of milk, millet, fruit juice and Medicine	(106)
*Lactococcus lactis*	Helps improve the texture and flavor profile of fermented products, and breaks down metabolic amino acids to produce volatile flavor substances	Cheese fermentation	(107)

## Mechanism of action of probiotics

### Enhancement of barrier function for intestinal mucosa

**Figure 6:**
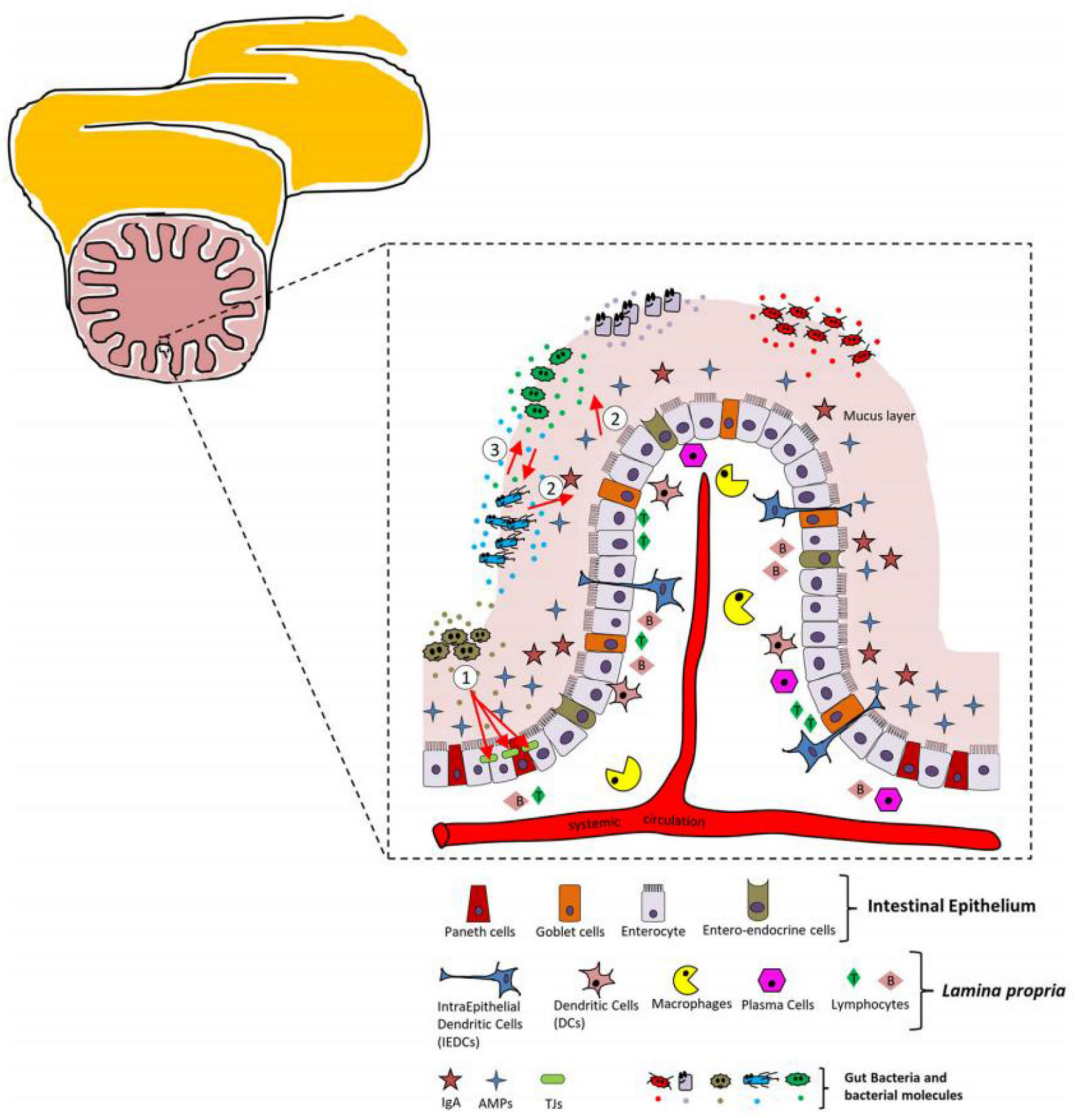
Schematic representation of the intestinal barrier and major cellular players.

As the largest immune organ in the body, intestine is very important to human body. The intestinal barrier is a heterogeneous body composed of extracellular components, including the mucus layer, the cellular layer and the lamina propria of the intestinal epithelium (8). Figure 6 presents relevant details of the intestinal barrier and the main cellular players.

Both mucus layer and intestinal epithelium possess the own specific cell types, which can be a physical barrier to intestinal microorganisms. For example, enterocytes can absorb the molecules from the intestinal lumen. Paneth cells possess the ability to synthesize and secrete antimicrobial peptides during contact with intestinal bacteria. *Saccharomyces cerevisiae* cells can secrete mucus. Intestinal endocrine cells are the constituent part of intestinal epithelium (72, 108, 109). The main roles of the intestinal epithelial mucus layer are to create a protective barrier against the hostile luminal environment, to facilitate the passage of food, and to avoid the adhesion of pathogens into the lamina propria (110).

Probiotics will interact with intestinal bacteria after entering the intestine; the first to play the role of physical barrier is the intestinal mucosa, which keeps the intestine at a safe distance from toxic substances in the intestinal lumen. Probiotics will react with bacteria after entering the intestine to enhance its chemical barrier, mechanical barrier, biological barrier and immune barrier (111). Reaching the intestine, probiotics will interact with intestinal cells, with the aim of restoring intestinal permeability, stimulating mucus production, promoting mucosal regeneration, and maintaining the mucosal barrier's integrity and the intestinal mechanical barrier's normal function (112). For example, some studies have found that antibiotics disrupt normal intestinal microbes and cause disruption of the intestinal barrier. However, the water-soluble polysaccharide from *Fagopyrum esculentum* Moench bee pollen can alleviate antibiotic-induced microbiota dysbiosis and improve intestinal barrier integrity by increasing intestinal sIgA secretion and suppressing inflammation (113).

### Enhancement of the immune response of the system

Certain probiotics in the gut can influence the function of various immune cells such as monocytes, macrophages, T cells, B cells and natural killer (NK) cells in the body in a direct or indirect way, thus acting as immune regulators and controlling inflammation; some of these probiotics are of the immunostimulatory type (9, 114, 115). These probiotics enhance non-specific cellular immune responses characterized by fighting against cancer cells, inducing IL-12 production, thus activating NK cells and developing Th1 cells, and releasing various cytokines in a strain-specific and dose-dependent manner; they also fight against allergies through the balance between Th1 and Th2 (116). To improve the intestinal mucosal immune system, for example, yogurt can be used to deliver the required probiotics to increase the number of IgA+ cells and cytokine-producing cells in the intestinal effector sites and enhance the immune response of the system (117). Short-term supplementation with probiotics can also enhance the body's cellular immune function. One study found that probiotics increased the body's polymorphonuclear phagocytosis capacity and tumor-killing activity of NK cells and improved cellular immune function in older people after supplementation with appropriate amounts of probiotics (118).

**Figure 7:**
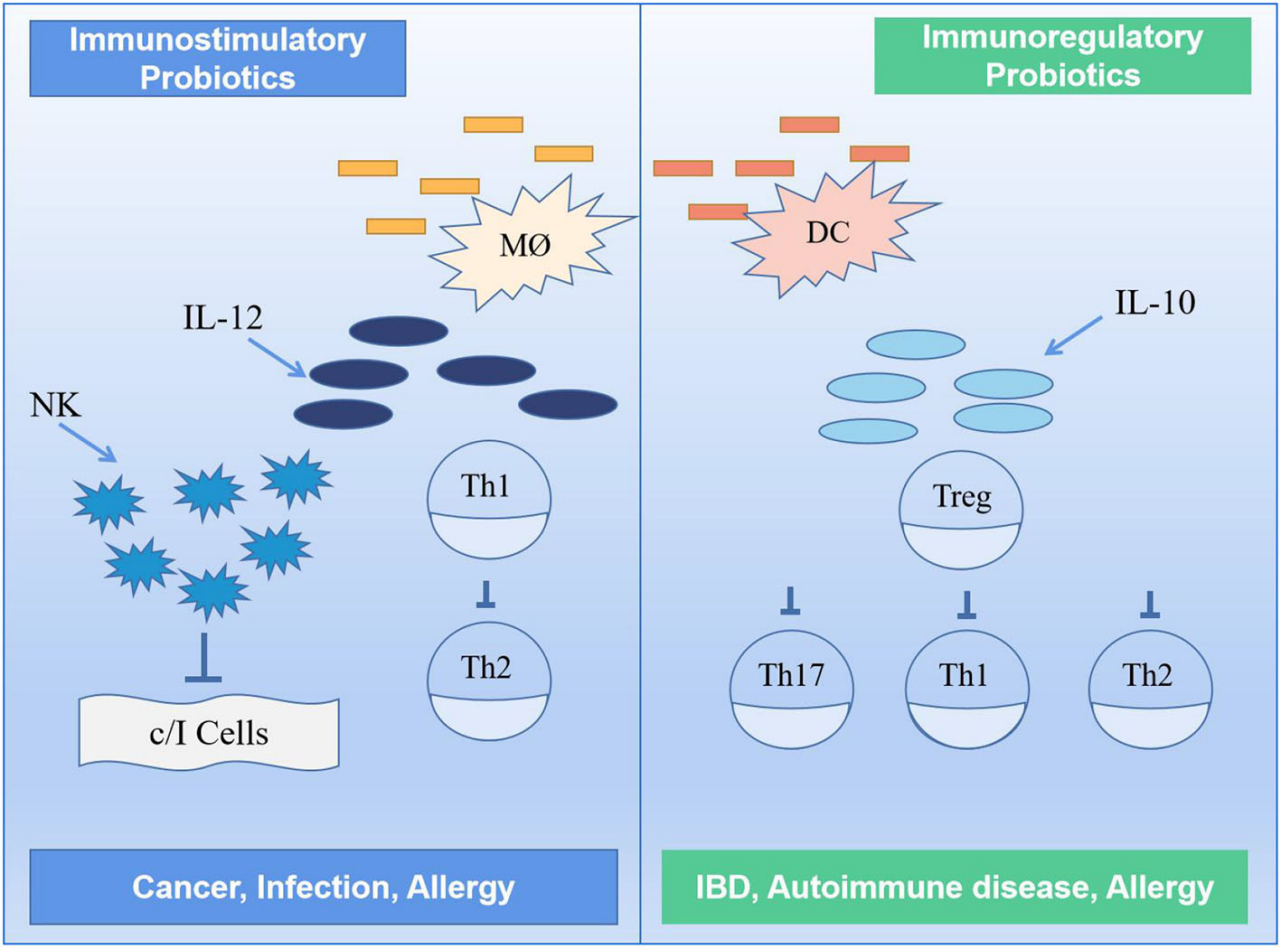
Mechanism of immune regulation by probiotics (117). TH1, TH2, TH16, type 1 T helper, type 2 T helper, type 17 T helper; DC, dendritic cell; MØ, M cell; IL-10, IL-12, interleukin-10, interleukin-12; NK, natural killer.

Generally, the probiotics can directly or indirectly stimulate immune cells in the gut to enhance its function. Some probiotics (i.e., immunomodulatory probiotics) can also regulate enzyme activity by altering microbial metabolism (119). If exposed to any foreign antigen, the host intestinal mucosal immune system initiates an immune response, partly through an adaptive immune response and partly by inducing inflammation to maintain homeostasis in the body. Immunomodulatory probiotics are characterized by the production of IL-10 and Treg cells, leading to a reduction in symptoms such as allergy and inflammation (120, 121). It has been found that probiotics increase intestinal barrier function by stimulating B cells and influencing cytokine production, thereby initiating an adaptive response in the host body, and that short-term supplementation with probiotics can also enhance the body's cellular immune function. In the presence of probiotics, monocytes act synergistically with NK cells to reduce and inhibit inflammatory cytokine release by secreting IL-10 to induce regulatory differentiation of stem cells and resistance to NK cell-mediated cytotoxicity (122). Figure 7 shows an immunomodulatory mechanism involving two different classes of probiotics, namely, immunostimulatory and immunomodulatory.

### Prevention of pathogenic bacteria adsorption and colonization

Probiotics regulate the intervention of intestinal microorganisms through colonization by modulating microorganism metabolism and improving human health. Probiotics prevent gastrointestinal diseases by competing for nutrients or producing antimicrobial factors to form colonization resistance to reduce infection by intestinal pathogenic microorganisms. The health of rabbits can be improved by using the autogenous strain *Enterococcus faecalis* EF2019 (CCM7420), which has a good ability to colonize the intestinal tract and produces the antimicrobial enterococin Ent7420, which effectively reduces coagulase-positive staphylococci, coliforms and clostridia (123). In addition to the gut, probiotics have the ability to colonize the epithelial surface and produce antimicrobial metabolites capable of controlling and maintaining the microbiota of the vagina. For example, *Lactobacillus acidophilus* KS400 produces bacteriocins with antimicrobial activity against relevant urogenital pathogens (124).

Because the activity of probiotics depends on the conditions of the host gastrointestinal tract and changes in the intestinal flora, the colonization and persistence of probiotics are important. Probiotic strains secrete secondary metabolites such as SCFAs and peptides with antimicrobial activity, which may interact directly with the host or pathogen to prevent proliferation of pathogens and improve the efficacy of probiotics (125). Probiotics can adsorb to mucus and epithelial cells to create a competitive advantage over pathogenic bacteria and bind to the host to produce stronger interactions and stimulate the host's immune response (126).

Thus, probiotics can be ingested as exogenous bacteria, colonize the human intestine, change the composition of intestinal flora, and then compete with pathogenic bacteria for nutrients, further excluding pathogenic bacteria and improving the immunity of the body.

### Mechanism of action between probiotics and gut-brain axis

The importance of the gut-brain axis in maintaining homeostasis in the body has long been appreciated, and it is considered a central nervous system pathway that regulates bidirectional communication between the gut and the brain at the neuronal, endocrine, and immune levels (127). As one of the important regulators of the gut-brain axis, microbiota have been of great interest in understanding of the importance of the gut-brain axis. Current evidence suggests that the gut microbiota and the brain communicate with each other through various pathways, including the immune system, tryptophan metabolism, the vagus and enteric nervous systems, and other mechanisms that may be involved in gut microbial signaling to the brain involving microbial metabolites such as SCFAs, branched-chain amino acids and peptidoglycans (128). In turn, the brain can change the composition and behavior of microorganisms through the autonomic nervous system (129, 130).

**Figure 8:**
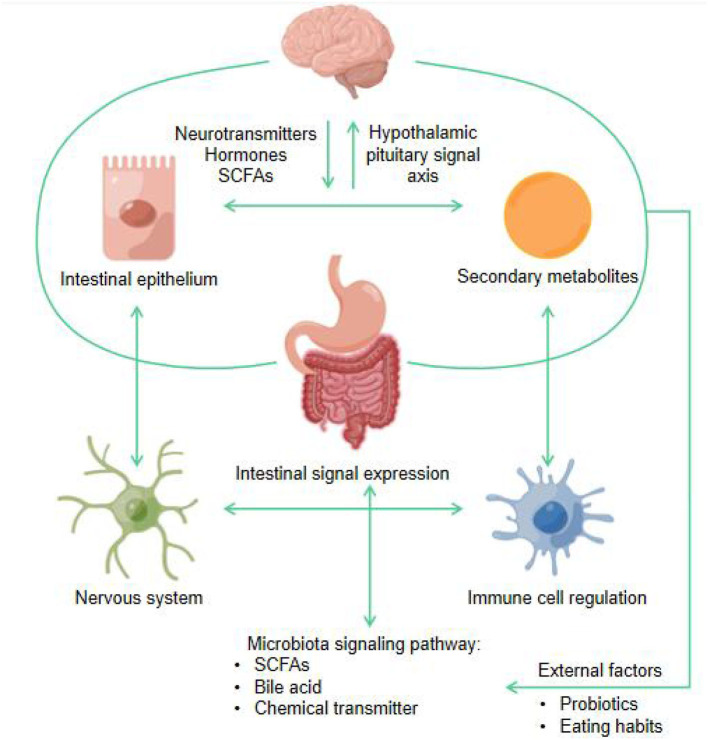
Schematic diagram of microbiota gut-brain axis bidirectional signal pathway (by Figdraw).

Many hormones and neurotransmitters are made in the gut, such as SCFAs, secondary products from carbohydrate fermentation, dopamine and serotonin, which can directly influence brain function and behavior (131). The gut microbiota broadly and profoundly affects the gut-brain relationship, including mental status, mood regulation, neuromuscular function, and hypothalamic-pituitary-adrenal (HPA) axis regulation, and the emotional and cognitive centers of the brain are affected either directly or indirectly (132). Figure 8 shows the schematic diagram of microbiota gut-brain axis bidirectional signal pathway.

The relationship between intestinal flora and depression has been a hot topic of research in recent years. The gut microbiota has been shown to be involved in the pathogenesis of depression, and although the relevant pathogenesis is unclear, it may be associated with modulation of monoamine neurotransmitter release and efficacy, altered activity and function of the HPA axis, and changes in the abundance of brain-derived neurotrophic factor. Therefore, attempts to target the microbiota-gut-brain axis to treat depression are increasing (132, 133). Studies have found that probiotics promote the production of SCFAs such as butyric acid, which is very important for the integrity of the intestinal barrier, it affects the central nervous system by changing the expression of BDNF and also has a positive impact on reducing the incidence rate of depression (134). To determine the effect of probiotic intake on depressive symptoms and metabolic status in major depressive disorder patients, Akkasheh et al. (135) conducted an experiment on 40 major depressive disorder patients (i.e., ingesting probiotic supplements composed of *Lactobacillus acidophilus, Lactobacillus casei*, and *Bifidobacterium bifidum*). They found that the administration of probiotic showed positive effect to decrease in the Beck Depression Inventory index and a significant decrease in insulin levels. This result is consistent with the experimental results of Kazemi et al., where, among 110 patients who were randomly treated with probiotics for 8 weeks, Beck Depression Inventory scores decreased significantly (136).

New research points to a link between autism and imbalance in the gut microbiota. Srikantha and Mohajeri (137) tested metabolites in the urine of children with autism and found that patients had abnormal levels of SCFAs, LPS, and indoles, which are likely to be caused by an incomplete gut barrier. Therefore, probiotics can be used to regulate the gut flora and re-establish gut homeostasis. The possible pathogenesis of Alzheimer's disease is like that of autism, where increased intestinal barrier permeability and immune cell activation impairs blood-brain barrier function, loses neurons, promotes neuroinflammation, and causes nerve damage, leading to disease onset. The use of probiotics as a supplement can regulate the balance of intestinal flora, which introduces a research direction for the treatment and prevention of Alzheimer's disease (138).

## Promotion mechanism of prebiotics for probiotics

### Promoting the growth and multiplication of probiotics

Previous studies have found that prebiotics can promote the growth of probiotics in the human gut and improve intestinal microbial diversity (139, 140). Probiotics account for a certain proportion of the normal intestinal flora in the intestine; they can inhibit the growth of harmful bacteria, regulate the human immune mechanism, and are closely related to human health. Probiotics cannot grow and metabolize without a carbon source (mainly carbohydrates). Prebiotics are not decomposed by human digestive juices, and indigestible prebiotics can be converted into carbon sources required by probiotics in the intestine, promoting the proliferation of good bacteria and regulating the composition of probiotics.

There are many studies on prebiotics promoting the growth and reproduction of probiotics, most of which focus on polysaccharide-based prebiotics. For example, Vázquez-Rodríguez et al. found that the polysaccharide fraction isolated from brown seaweed *Silvetia compressa* could proliferate *Bifidobacterium* and *Lactobacillus* to increase the synthesis of total SCFAs with effects similar to inulin (141). Shang et al. (142) investigated *Enteromorpha clathrata* polysaccharide and found that *Enteromorpha clathrata* polysaccharide greatly altered the structure of the intestinal microbiota and significantly promoted the growth of probiotics in C57BL/6J mice. Moreover, *Enteromorpha clathrata* polysaccharide affected the microbiota differently in females and males, with *Enteromorpha clathrata* polysaccharide causing an increase in the number of *Lactobacillus* bacteria in male mice and *Bifidobacterium* and *Akkermansia muciniphila* (the next generation of probiotics) in females. The positive effect of sulfated polysaccharides isolated from sea cucumber *Stichopus japonicus* (SCSPsj) on rodent health was attributed to the significant modulation of the gut microbial community by SCSPsj due to the regulation of gut microorganisms. Although not directly proliferating LAB, SCSPsj significantly promoted biofilm formation and mucus binding, contributing to their enrichment *in vivo* and indirectly increasing their abundance (143).

In addition to polysaccharide prebiotics, polyphenolic compounds can selectively promote the growth of probiotics. The existed phenolic compounds (e.g., catechin, gallic acid, vanillic acid, and protocatechuic acid) in mangos can inhibit the growth of pathogenic bacteria and have a good effect on the growth and reproduction of probiotics (144). Not only limited to the intestinal tract, *Sphallerocarpus gracilis* polysaccharides both enhance the acidifying activity of *Streptococcus thermophilus, Lactobacillus plantarum* and *Lactobacillus rhamnosus* (which is termed as *Lacticaseibacillus rhamnosus* according to the updated microbial taxonomy) during milk fermentation and promote the growth of these probiotics (145). Overall, the above findings are a good indication that prebiotics can be utilized by certain probiotics, thus increasing flora abundance.

### Promoting the metabolism of probiotics

SCFAs are the metabolism products of fermentation of carbohydrates and proteins by intestinal bacteria from endogenous or dietary sources. They consist of saturated fatty acids with chains of 2–6 carbons (146). Probiotics and prebiotics are the most important factors in the formation of SCFAs. In the intestine, prebiotics produce beneficial metabolites in the presence of probiotics, predominantly SCFAs, which influence the intestinal environment and lower intestinal pH (147).

SCFAs are mostly produced by anaerobic bacterial fermentation of undigested and unabsorbed carbohydrates in the colon, with SCFAs-producing probiotics mainly including *Lactobacillus, Bifidobacterium* and *Clostridium Butyricum*. Prebiotics have an important effect on SCFAs production by probiotics. One study showed that the physical form of the prebiotic substrate affected fermentation rate and SCFAs production (148). Higher crystallinity bacterial cellulose had lower fermentation rates than less crystalline soluble polysaccharides, and cellulose complex fermentation produced a different SCFAs profile compared to soluble polysaccharides, with significantly higher butyric acid production and lower propionic acid production than that of rapidly fermentable substrates (149).

**Figure 9:**
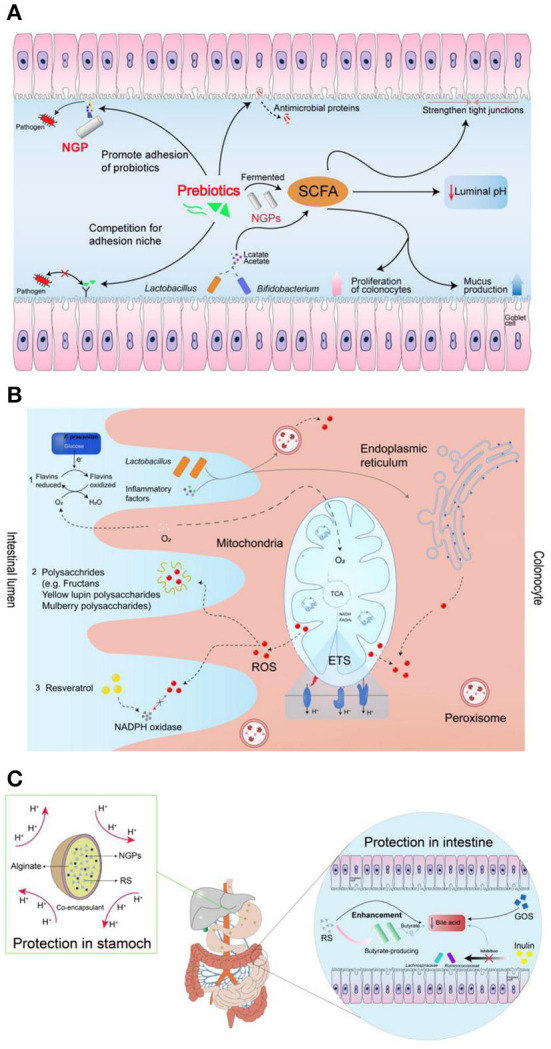
The mechanism of prebiotics promoting probiotics: **(A)** prebiotics for enhancing the functions of probiotics; **(B)** prebiotics for enhancing the resistance of probiotics to reactive oxygen species; **(C)** protective functions of prebiotics by maintaining the viability of probiotics during gastrointestinal transit (154).

The concentration of prebiotics is also an important factor affecting the amount SCFAs produced by probiotics. Fehlbaum et al. found that five prebiotics of varied concentrations (i.e., inulin, α-GOS, β-GOS, xylo-oligosaccharides and β-glucan) to explore their regulatory effects on intestinal microorganisms (150). The experimental results showed that β-glucan had the most significant effect on microbial composition and metabolism—as it promoted the growth and reproduction of *Prevotella* and *Roseburia*—resulting in a significant increase in the production of propionic acid. In addition, the proportion of butyrate increased with the increase of β-glucan and inulin concentrations. As an important energy source for intestinal epithelial cells, butyrate has beneficial properties such as anti-colon cancer and anti-inflammation effects (151, 152). Taking tea dietary fiber as an example, it was found that with the increase of tea dietary fiber concentration, the pH value in the fermentation broth could be significantly reduced, where 3% dietary fiber powder showed the most significant effect (153). In addition, Fei et al. (154) found that prebiotics could be fermented into the SCFAs, which showed the benefits to promote the niche opportunity of probiotics by strengthening tight junctions, promoting colon cell proliferation and mucus production, and lowering intestinal pH. The produced SCFAs can also be regarded as the nutrients to enhance the function of probiotics (155). In addition, prebiotics and pathogens can competitively bind to the receptors on epithelial cells, facilitating the probiotics to produce antimicrobial peptides to show the antibacterial effect. The detailed mechanisms of prebiotics for enhancing the functions of probiotics were shown in Figure 9A (154).

In general, prebiotics promote the production of SCFAs (mainly butyric acid, propionic acid, and acetic acid) by probiotics, and the increase in SCFAs also leads to a decrease in the pH of the intestine and is beneficial to human health. In the human intestine, a suitable pH value is conducive to maintaining the adhesion ability of probiotics and promoting their growth and colonization, and a lower intestinal pH value can effectively inhibit the reproduction of harmful bacteria and promote the reproduction of probiotics.

### Promoting the resistance of probiotics to reactive oxygen species and bile salts/acids

It is well known that the existing bacteria and probiotics in the intestine survive under the oxidizing environment with oxygen and reactive oxygen species (ROS), which is produced from the steps of tricarboxylic acid (TCA) cycle and the electron transport system (ETS) during the process of oxidative phosphorylation of oxygen in mitochondria (156). The moderate amount of ROS shows benefits to keep the health of the human body. While, the excessive amount of ROS in the intestine can induce an adverse effect on the diversity and survival of intestinal probiotics by reacting with DNA, proteins, and lipids in their cell membranes (157).

It has been reported that the prebiotics of fructans, plant polyphenols, inulin, and yellow lupin polysaccharides could scavenge the free ROS in the gastrointestinal tract, which showed the protective effect for probiotics (158–160). The capability of prebiotics to scavenge ROS is due to the produced butyrate acid in SCFAs can consume the oxygen during the metabolism process in the gut. As shown in Figure 9B, the decreased oxygen concentration further can adjust the habitability of gut environment for oxygen-sensitive probiotics (154, 161).

Generally, bile shows the ability to promote the digestion and absorption of lipids in the body. Most probiotics in the intestine are extremely sensitive to bile salts/acids, which can inhibit the growth and function of probiotics by causing oxidative stress, dissolving bacterial membranes and damaging DNA (162, 163). It is reported that bile salts/acids can be degraded by the prebiotics of GOS, resistant starch (RS), and inulin-type fructans *via* binding up bile acids, reducing their reabsorption and increasing their turnover rate in the intestines (164). For the probiotics, they can be incorporated into microcapsules with the wall materials of used prebiotics, which can avoid exposure of probiotics in the gastric fluids. In addition, the produced butyrate by prebiotics shows an ability to reduce the toxicity of bile salt by reversing the hyper proliferation of the colonic surface induced by deoxycholate (165). The potential protective functions of prebiotics by maintaining the viability of probiotics during gastrointestinal transit was shown in Figure 9C (154).

### Utilization of prebiotics by probiotics is related to the degree of polymerization of the former

The DP of prebiotics is closely related to probiotic activity. In general, the lower the prebiotic DP, the stronger the prebiotic effect, and the easier it is to be used by probiotics, the possible reason being that prebiotics of lower molecular weight are thought to have active groups that are more exposed. To investigate the effects of fibers with different degrees of polymerization on human intestinal bacteria, Chen et al. (166) investigated the pH, air pressure, and SCFAs content of fecal fermentation with three fiber substrates of different DPs (i.e., carboxymethylcellulose, β-glucans, and GOS). The results showed that operational taxonomic units in *Bifidobacterium, Streptococcus*, and *Lactobacillus* were then negatively correlated with DP, indicating that lower values of fiber DP produced greater probiotic effects.

As the most common prebiotic, the polymerization degree of inulin has also been widely studied. The prebiotic effect of inulin mainly depends on its DP, which determines its degradation site, hydrolysis rate and fermentation products. Li et al. (167) investigated the effect of inulin with different DP on the intestinal microbiota of c57bl/6 J mice fed a HFD. The experiment found that short-chain inulin preferentially stimulated *Bifidobacterium*, which showed that *Bifidobacterium* had the ability to effectively use oligosaccharides. Besides, inulin may inhibit the secretion of endotoxin by increasing the proportion of *Bifidobacterium* and *Lactobacillus*, which is conducive to anti-inflammatory activity. Interestingly, long-chain inulin preferentially stimulates the growth of *Bacteroides*, which has a series of enzymes that can degrade complex polysaccharides into oligosaccharides and monosaccharides. This finding also shows that long-chain inulin is more dependent on bacteria (such as Bacteroides) that can process complex polysaccharides than short-chain inulin, as inulin with a high DP must be hydrolyzed to monosaccharides before being used by bacteria. Zhu et al. (168) proved that in terms of stimulating probiotics, inulin intervention measures with low DPs were more effective than those with high DPs. Specifically, the increase of *Lactobacilli, Bifidobacteria* and *Myxomycetes* in the FOS group was higher than that in the inulin group; because the DP value of inulin was higher than that of FOS, it was easier to hydrolyze into monosaccharides and be digested and utilized by probiotics, which also showed that compared with inulin with a high DP, inulin with a low DP ferment faster in the intestinal microbiota *in vitro*. In addition, this study also showed that the dosage of prebiotics affected the increase in probiotics. At high doses, the increase in probiotics' number was more excellent.

From the above research, it can be concluded that inulin with a low DP has a more obvious impact on the structure of intestinal flora than that with a high DP. The reason may be related to the water solubility of inulin. Generally, the water solubility of oligomeric inulin is higher than that of high-aggregation inulin. Short-chain inulin is easier to dissolve in water than long-chain inulin, which is conducive to the rapid utilization of probiotics.

### As the protective agent in probiotic viable preparations

Probiotics have a variety of functions and can be used as supplements for human or animals. They are widely used in food, drugs, cosmetics, health products, feed and other fields. In order to increase the longevity of probiotics, it is best to dry them (169). In the preparation of probiotics, they are usually dried into powder by freeze-drying or spray drying. However, adverse environmental conditions, such as acid, heat, pressure, and oxygen, can also cause a significant decline in the cell viability of probiotics (170).

Freeze-drying can protect the probiotic from external invasion during storage, maintain the properties and bacteria number of probiotic powders, and give better play to the probiotic effect. This method has the advantages of convenient transportation, maintaining bacteria activity, and long-term storage (171). However, due to the influence of various factors in the freeze-drying process, the bacteria may die. Therefore, a protective agent can be used to change the environment of probiotics during freeze-drying, reduce the damage to cells, and maintain the original physiological and biochemical characteristics and biological activities of microorganisms as much as possible (172).

A prebiotic is one of the commonly used protective agents. Savedboworn et al. (173) studied protein-trehalose as a protective agent and found that it has a significant impact on the survival of probiotic *Lactobacillus plantarum* TISTR 2075 grown in the extract of Plai-Ngahm-Prachinburi rice and improved the survival rate of the strain after freeze-drying (98.13%). Compared with other protective agents, even under long-term storage, the protein-trehalose protective agent maintained a high number of living cells and the lowest cell death rate. Shu et al. (174) optimized the compound cryoprotectant containing lactose (21.24%), trehalose (22.00%), and sodium glutamate (4.00%). The optimized protective agent exhibited an *S. boulardii* survival rate as high as 64.22%, and a maximum number of living cells of 9.5 × 10^9^ cfu/g, which proved that the protective agent prepared from prebiotics had a good protective effect on probiotics during freeze-drying.

Another study evaluated the potential of spent brewer's yeast β-glucan (YβG) as a protective agent for probiotic *Lactobacillus* cultures and compared it to two common prebiotic protectors, FOS and oligofructose. The experimental results showed that β-glucan and FOS protected *Lactobacilli* similarly during the first 90 days of refrigeration (4°C). However, after 90 days, FOS provided higher protection and resulted in lower cell membrane damage. In contrast, for *Lactobacillus plantarum* 201, YβG was more effective as a protective agent (175). It is worth noting that for different probiotic species, the effect of different prebiotics as protectants varied, indicating that the effect of cryoprotectants varied with the strains tested.

Compared with freeze-drying, spray-drying takes less time, consumes less energy, costs less, and is more suitable for industrial production (176, 177). The process of spray drying cannot avoid of drying and dehydrating at high temperatures, which will destroy the structure of proteins and nucleic acids, and disrupt the connection between monomer units, largely affecting the viability of probiotics. Therefore, protective agents play a crucial role in protecting probiotics from adverse conditions and in storing them for long periods after drying (170).

For example, Verruck et al. (178) investigated how to improve the survival of *Bifidobacterium* BB-12 and found that using goat milk (200 g L^−1^) and inulin (100 g L^−1^) as carriers, the survival rate of *Bifidobacterium* is higher than that without or with FOS, and the spray dried powder produced has the lowest water activity and the best powder stability. Similar results were obtained by Dantas et al., whose experimental results illustrated that the use of inulin as a protective agent was more effective than that of no using or use of FOS, with an encapsulation rate of 88.01% and the highest survival rate of *Bifidobacteria* during 120 days of storage (179). Both of these studies illustrate the advantages and potential of prebiotics as a protective agent in the preparation of live probiotic formulations, which can effectively improve the survival of the target strains and maintain good viability during storage.

## Summary and prospective

In the current study, prebiotics and probiotics have shown excellent ability to regulate human health, especially the balance of intestinal microorganisms. When they are applied in health food, clinical and other fields, they can show excellent health effects of preventing some diseases, regulating human health, secreting or synthesizing beneficial substances such as antibiotics and SCFAs, and increasing the number of beneficial bacteria. However, further research should be carried out. First, the current mechanism of prebiotic selective promotion of probiotics remains to be explored, especially whether the concentration, preparation method and glycosidic bond connection form of prebiotics have an impact on the utilization efficiency of probiotics, which would aid in selecting the appropriate prebiotics to promote probiotics in practical applications. Second, the mechanism of prebiotics entering the intestinal flora needs to be further revealed. Although many studies have proved the promoting effect of prebiotics on the intestinal flora, the experimental methods and theoretical mechanism still need further improvement. Third, the influence and mechanism of probiotics on the gut-brain axis need to be further explored, which is also an important direction of future development.

## Author contributions

SY and YM conducted the literature search and wrote the first draft of the manuscript. BY and WP draft the figures in the manuscript. QW, CD, and CH revised the manuscript. All authors have read and agree to the published version of the manuscript.

## Funding

This work was sponsored by the Jiangsu Qing Lan Project and the Young Elite Scientists Sponsorship Program by CAST for CH. In addition, the authors thank the Fuzhou Science and Technology Project (AFZ2021K010003), the Fujian Key Laboratory of Inspection and Quarantine Technology fund (FJKF2021-01), the Opening Project of Key Laboratory of Coarse Cereal Processing of Ministry of Agriculture and Rural Affairs, Chengdu University (No. 2022CC010) and the Cooperation Project with ChenXing (No. R-2022-H-002MF) to support this work.

## Conflict of interest

Author QW was employed by Nutrilite Health Institute. The remaining authors declare that the research was conducted in the absence of any commercial or financial relationships that could be construed as a potential conflict of interest.

## Publisher's note

All claims expressed in this article are solely those of the authors and do not necessarily represent those of their affiliated organizations, or those of the publisher, the editors and the reviewers. Any product that may be evaluated in this article, or claim that may be made by its manufacturer, is not guaranteed or endorsed by the publisher.
